# Editorial: Functional neuroendocrine tumors

**DOI:** 10.3389/fendo.2025.1662247

**Published:** 2025-07-23

**Authors:** Bojana Popovic, Francesca Spada, Aviral Singh, Karin Zibar Tomsic

**Affiliations:** ^1^ Clinic for Endocrinology, Diabetes and Metabolic Diseases, University Clinical Centre of Serbia, Belgrade, Serbia; ^2^ Faculty of Medicine, University of Belgrade, Belgrade, Serbia; ^3^ European Institute of Oncology, IEO, IRCCS, Milan, Italy; ^4^ Theranostics (Oncology), GenesisCare Australia Pty Ltd, Perth, WA, Australia; ^5^ Department of Nuclear Medicine, Fiona Stanley Hospital, South Metropolitan Health Service, Perth, WA, Australia; ^6^ Department of Endocrinology and Diabetology, University Hospital Centre Zagreb, Zagreb, Croatia

**Keywords:** functional NET, neuroendocrine tumor, rare tumors, hormonal secretory syndrome, advances in diagnosis and therapy

One of the authentic features of neuroendocrine tumors (NETs) is a potential to synthesize and secrete biogenic peptides and hormones, which can cause recognizable clinical hormone syndromes. These functional NETs are a minority compared to non-functional tumors (i.e. 10-40% in a group of pancreatic NETs), yet they pose significant diagnostic and therapeutic challenges (1). In any case of functional NET, hormonal hypersecretion syndrome leads to excessive morbidity, sometimes more threatening than the malignancy itself (2). Despite the advances in diagnostics and therapy, many issues are still unresolved. This Research Topic aims to address the emerging diagnostic and management strategies, some of which point to the specific biology of these tumors.

At present, the diagnosis of functional NETs relies on clinical presentation and corresponding hormonal secretory profile. Bolduan et al. bring an entirely new perspective by analyzing the expression of sortilin (neurotensin receptor 3), a transmembrane receptor involved in intracellular trafficking of various proteins. Sortilin is associated with several metabolic, neurologic and inflammatory conditions, and has also been shown to contribute to tumorigenesis by promoting cell adhesion and migration (3, 4). Bolduan et al. demonstrate that sortilin expression is twice as high in functional compared to non-functional NETs of different primary origins. This positions sortilin as a potential biomarker for functional NETs, but also as a therapeutic target since the inhibition of sortilin reduced cellular serotonin concentrations in serotonin-producing cell lines.

From a diagnostic and therapeutic standpoint, the expression of somatostatin receptors, particularly type 2 (SSTR2), in various types of NETs is crucial for both localization and treatment with radiolabeled or synthetic somatostatin analogues. The expression of SSTR2 is under negative control of glucocorticoids (5), which makes functional diagnostics and treatment challenging in patients with Cushing’s syndrome due to ACTH secreting NET. Pivonello et al. tested the ability of a highly selective non-steroidal GR modulator relacorilant to overcome glucocorticoid induced suppression of SSTR2. In an experimental model of murine At-T20 cell line relacorilant inhibited dexamethasone-mediated reduction of SSTR2A/2B mRNA in a concentration-dependent manner. The authors also demonstrated upregulation of SSTR2 with relacorilant in 4 patients with ACTH-secreting NETs (2 patients with ectopic ACTH secreting NETs (2 patients with ectopic Cushing’s syndrome due to ACTH-secreting lung NETs, and 2 patients with Cushing’s disease due to ACTH-secreting pituitary adenoma).

Given the high rate of surgical cure, preoperative localization of insulinoma is of utmost importance. However, this can be very challenging as these tumors often measure less than 2 cm, may be multifocal, and are sometimes associated with nesidioblastosis. In cases of diagnostic failure with standard morphological imaging (contrast-enhanced CT, MRI and EUS), functional imaging with ^68^Ga-DOTA-exendin-4-PET/CT has proven to be the most sensitive method, due to the high expression of GLP-1 receptors in benign insulinomas (6). Since the availability of this method is very limited, and sensitivity of ^68^Ga-DOTA-SSA or ^18^F-DOPA PET insufficient, intraarterial calcium stimulation with venous sampling (ASVS) can be utilized as a highly sensitive and specific alternative. Halmi et al. present institutional experience with ASVS in preoperative localization of insulinoma, providing a detailed methodology and interpretation of results in 9 patients. The authors provide a detailed comparison of ASVS with other methods for localization of insulinoma.

As most insulinomas are benign solitary tumors, minimally invasive procedures have emerged as optimal approaches, offering clinical success with fewer adverse events compared to classical open surgery. These include not only minimal invasive surgery (MIS) but also interventional procedures like EUS-guided radiofrequency ablation (RFA) (7). In a meta-analysis, Xiao et al. compared MIS to EUS-guided ablation. Even though the latter had higher recurrence rates, it was associated with lower adverse event rates and shorter hospital stays, making it a viable option for poor surgical candidates.

Since the first report in 2001 (8), only a few other publications presented the use of RFA in the treatment of pheochromocytoma/paraganglioma (PPGL), either for metastatic lesions or small inoperable primary tumors. Magalhaes et al. bring an additional perspective on the utilization of RFA by treating a large, locally aggressive and functional retroperitoneal PGL. Performed by an experienced team, the procedure resulted in a remarkable overall tumor, clinical and biochemical responses. The authors also provide an overview of other potential treatment options for patients who are unsuitable for surgery.

Peptide receptor radionuclide therapy (PRRT) has rarely been examined exclusively in functional NETs. Unlike some anticancer therapies, PRRT has both antiproliferative effect and provides symptom control through reduction in hormonal secretion (9, 10). Nonetheless, certain studies suggest worse overall response rate in functional NETs compared to non-functional tumors (11). In a retrospective study of 51 patients with NETs of various primary locations, Vukomanovic et al. identified tumor functionality as the strongest predictor of poorer survival. By analyzing a large group of 447 pancreatic neuroendocrine neoplasm (pNEN) patients treated with PRRT, Singh et al. developed a clinicopathological and imaging parameter-based nomogram (PANEN-N) for predicting overall survival following PRRT in patients with pNEN. Tumor functionality was shown to be one of the negative predictors of survival.

The final segments of this Research Topic present rare types of functional NETs. Zhao et al. describe an extremely rare case of ectopic ACTH secreting appendiceal NET. By analyzing this patient and 7 other cases reported in the literature so far, the authors thoroughly describe all diagnostic pitfalls regarding this entity.

As much as any case of ectopic Cushing’s syndrome can lead to an unnecessary pituitary surgery, this risk is even greater with ectopic acromegaly. Zendran et al. present an extensive review of 127 published cases of ectopic acromegaly (mostly originating from the lung and the pancreas) covering a broad range of clinical characteristics. Since it can be extremely challenging to differentiate pituitary from ectopic acromegaly solely based on clinical presentation, the authors focus on all diagnostic difficulties and present therapeutic options.

Nguyen et al. give a presentation of an extremely rare type of paraganglioma located in the urinary bladder (UBPGL). These rare tumors can present with symptoms and signs of catecholamine excess that are characteristically triggered by micturition. In addition to describing diagnostic approach and surgical treatment options, the authors give a detailed methodology and interpretation of molecular testing for both germline and somatic pathogenic variants that cause paraganglioma.

Lorenço et al. draw attention to insulinomatosis, a rather novel entity that causes persistent hyperinsulinemic hypoglycemia. As in the first formal description in 2009, insulinomatosis represents multiple micro- and macrotumors as well as cell clusters that exclusively produce insulin and arise in the pancreas without genetic background (i.e. syndrome of multiple endocrine neoplasia type 1 – MEN1) (12). The authors present four cases in detail, emphasizing the morphological/immunohistochemical specificities of the disease, and the challenges of achieving surgical cure.

Finally, Cidade-Rodrigues et al. present another rare pancreatic endocrine disorder that only recently reemerged in the literature, which is alpha-cell hyperplasia (ACH). The authors give an informative overview of three types of ACH (reactive, functional and non-functional) and explain differences in their clinical presentation, blood glucagon levels and in the potential to develop into pancreatic NETs. They also present an illustrative case of non-functional ACH with glucagon-producing PNET.

We hope that this Research Topic will be a valuable addendum to current knowledge regarding NETs, and that it will inspire future research.
